# Management of HIV-associated tuberculosis in resource-limited settings: a state-of-the-art review

**DOI:** 10.1186/1741-7015-11-253

**Published:** 2013-12-02

**Authors:** Stephen D Lawn, Graeme Meintjes, Helen McIlleron, Anthony D Harries, Robin Wood

**Affiliations:** 1 Department of Clinical Research, Faculty of Infectious and Tropical Diseases, London School of Hygiene and Tropical Medicine, Keppel Street, London WC1E 7HT, UK; 2 The Desmond Tutu HIV Centre, Institute for Infectious Disease and Molecular Medicine, Faculty of Health Sciences, University of Cape Town, Cape Town, South Africa; 3 Department of Medicine, Faculty of Health Sciences, University of Cape Town, Cape Town, South Africa; 4 Clinical Infectious Diseases Research Initiative, Institute of Infectious Disease and Molecular Medicine, University of Cape Town, Cape Town, South Africa; 5 Division of Clinical Pharmacology, Department of Medicine, Faculty of Health Sciences, University of Cape Town, Cape Town, South Africa; 6 International Union against Tuberculosis and Lung Disease (The Union), Paris, France

## Abstract

The HIV-associated tuberculosis (TB) epidemic remains a huge challenge to public health in resource-limited settings. Reducing the nearly 0.5 million deaths that result each year has been identified as a key priority. Major progress has been made over the past 10 years in defining appropriate strategies and policy guidelines for early diagnosis and effective case management. Ascertainment of cases has been improved through a twofold strategy of provider-initiated HIV testing and counseling in TB patients and intensified TB case finding among those living with HIV. Outcomes of rifampicin-based TB treatment are greatly enhanced by concurrent co-trimoxazole prophylaxis and antiretroviral therapy (ART). ART reduces mortality across a spectrum of CD4 counts and randomized controlled trials have defined the optimum time to start ART. Good outcomes can be achieved when combining TB treatment with first-line ART, but use with second-line ART remains challenging due to pharmacokinetic drug interactions and cotoxicity. We review the frequency and spectrum of adverse drug reactions and immune reconstitution inflammatory syndrome (IRIS) resulting from combined treatment, and highlight the challenges of managing HIV-associated drug-resistant TB.

## Introduction

The global epidemics of HIV/AIDS and tuberculosis (TB) both remain huge challenges to international public health, causing illness and death in millions of people worldwide each year (Table 1) [1]. TB is the most important AIDS-related opportunistic disease globally and is the leading cause of HIV/AIDS-related mortality, accounting for an estimated 25% of such deaths [2,3]. Sub-Saharan Africa suffers disproportionately, with 79% of global cases of HIV-associated TB [1]. In the countries of southern and eastern Africa where HIV prevalence is highest, the impact of HIV has severely undermined TB control over the past 20 years [4]. The global co-epidemic has been further compounded in recent years by the emergence of the growing challenge of multi-drug resistant TB (MDR-TB) [5,6].

**Table 1:** Burden of HIV infection, tuberculosis (TB) and HIV-associated TB globally and in sub-Saharan Africa

**Disease**	**Global burden**	**Burden in sub-Saharan Africa: (% of global burden)**
**HIV/AIDS**:		
No. of people living with HIV infection	34,200,000	23,500,000 (69%)
HIV/AIDS-related deaths	1,700,000	1,200,000 (71%)
**Tuberculosis**:		
No. of incident cases of TB	8,700,000	2,300,000 (26%)
TB deaths (excluding HIV)	990,000	220,000 (22%)
Incident cases of multidrug-resistant TB	310,000	45,000 (15%)
**HIV-**a**ssociated** t**uberculosis**:		
No. of incident cases	1,100,000	870,000 (79%)
No. of HIV-associated TB deaths	430,000	300,000 (70%)

Data from [1,3]. Incident disease and deaths represent annual disease burden.

The World Health Organization (WHO) DOTS (directly observed treatment, short-course) TB control strategy used in isolation provides far from optimum case management for individual patients with HIV-associated TB and it has failed to control TB at a population level in settings with high HIV prevalence [2,7]. Comprehensive packages of additional interventions are needed to address the consequences of HIV in TB patients and to reduce the burden of TB in those living with HIV infection [8]. An interim policy on collaborative TB/HIV activities was first published by WHO in 2004 [9] and approximately 1.3 million lives are estimated to have been saved by these interventions by 2011 [1]. An updated policy (Table 2) [10] published in 2012 provides the overall policy framework for addressing HIV-associated TB and specific recommendations on management of HIV, TB and multidrug-resistant (MDR)-TB are provided by individual guideline documents [11-13] (Table 3).

**Table 2:** **World Health Organization (WHO)-recommended collaborative tuberculosis (TB)/HIV activities (adapted from**[10]**)**

**Key area**	**Points of action**
**Establish and strengthen the mechanisms for delivering integrated TB and HIV services**	Set up and strengthen a coordinating body for collaborative TB/HIV activities
	Determine the HIV prevalence among TB patients and the TB prevalence among HIV patients
	Carry out joint TB/HIV planning to integrate the delivery of TB and HIV services
	Monitor and evaluate collaborative TB/HIV activities
**Reduce the burden of TB in people living with HIV (early ART plus the three Is)**	Intensify TB case finding and ensure high quality TB treatment
	Initiate TB prevention using isoniazid preventive therapy and early antiretroviral therapy (ART)
	Ensure control of TB infection in healthcare facilities and congregate settings
**Reduce the burden of HIV in patients with diagnosed TB and those under investigation for TB**	Provide HIV testing and counseling to both groups of patients
	Provide HIV preventive interventions to both groups of patients
	Provide co-trimoxazole preventive therapy for TB patients living with HIV
	Provide HIV prevention interventions, treatment and care for TB patients living with HIV
	Provide antiretroviral therapy for TB patients living with HIV

**Table 3:** World Health Organization (WHO) policy guidelines on collaborative tuberculosis (TB)/HIV activities and the management of HIV infection, TB and multidrug-resistant TB (MDR-TB)

**Guidelines/year**	**Details**	**Reference**
**Guidelines for collaborative TB/HIV activities (2012)**	World Health Organization. WHO policy on collaborative TB/HIV activities. Guidelines for national programmes and stakeholders. 2012. World Health Organization, Geneva. WHO/HTM/TB/2012.1. http://whqlibdoc.who.int/publications/2012/9789241503006_eng.pdf	[10]
**Antiretroviral treatment guidelines (2013)**	World Health Organization. Consolidated guidelines on the use of antiretroviral drugs for treating and preventing HIV infection. Recommendations for a public health approach, June 2013. WHO, Geneva. Accessible at: http://apps.who.int/iris/bitstream/10665/85321/1/9789241505727_eng.pdf.	[11]
**Tuberculosis treatment guidelines (2010)**	World Health Organization. Treatment of tuberculosis: guidelines - fourth edition. World Health Organization, Geneva, 2010. WHO/HTM/TB/2009.420 Accessible at: http://whqlibdoc.who.int/publications/2010/9789241547833_eng.pdf	[12]
**Drug-resistant TB treatment guidelines (2011)**	World Health Organization. Guidelines for the management of drug-resistant tuberculosis: 2011 update. WHO, Geneva. WHO/HTM/TB/2011.6. Accessible at: http://whqlibdoc.who.int/publications/2011/9789241501583_eng.pdf.	[13]

This article provides an up-to-date review of the current medical management of adult patients with HIV-associated TB. We review case ascertainment as the critical first step and then how clinical outcomes can be optimized by provision of effective TB treatment, use of concurrent ART, prevention of HIV-related comorbidities and management of drug cotoxicity and immune reconstitution inflammatory syndrome (IRIS). We also describe the management of HIV-associated MDR-TB. However, the management of children, models of integrated TB and HIV care delivery and prevention of TB in people living with HIV using ART and isoniazid preventive therapy lie outside the scope of this review.

### Diagnosis of HIV-associated TB

The prerequisite for optimum management of HIV-associated TB is early and accurate diagnosis and, for many years, this has been a key obstacle. Case ascertainment can be greatly improved by high rates of quality-assured HIV testing among those being investigated for TB as well as high rates of screening for TB in those living with HIV.

#### Screening for TB in those living with HIV infection

In high burden settings, much prevalent TB disease remains ‘below the radar’ in those living with HIV. Postmortem studies conducted in hospitals across sub-Saharan Africa over the past 20 years have repeatedly shown that between 30% and 50% of HIV-infected adult inpatients who die have postmortem evidence of TB, much of which was neither clinically suspected nor diagnosed before death [14-17]. These studies have highlighted the abject failure of the diagnostic process and the low sensitivity of diagnostic tools available [18]. In the absence of more sensitive means of diagnosis, management algorithms for suspected sputum smear-negative disease were developed [19-21] and studies of empirical TB treatment for certain high risk patient groups with advanced immunodeficiency are being conducted [22].

However, in recent years there have been significant advances in screening and diagnosis. Traditional symptom screening for pulmonary TB based on chronic cough has low sensitivity for HIV-associated TB [23,24]. A new WHO symptom screening tool for HIV-associated TB (one or more of the following symptoms: cough, fever, weight loss or night sweats, each of any magnitude or duration) has much higher sensitivity and is recommended for routine screening of those in HIV care at each visit [25]. However, in view of its low specificity, further research is needed to define which of the large number of patients with a positive screen should be prioritized for subsequent microbiological testing of clinical samples.

New diagnostic tools have also increased our capacity for microbiological diagnosis. This includes the Xpert MTB/RIF assay, which was endorsed by WHO in 2010. A single test is able to detect all sputum smear-positive disease, approximately 70% of smear-negative pulmonary disease and provides rapid simultaneous screening for RIF resistance [26]. In addition, this assay can be used to test a wide range of extrapulmonary sample types [26,27]. The Xpert MTB/RIF assay has been incorporated into the national guidelines of many high burden countries. In South Africa, which alone accounts for approximately 30% of the global burden of HIV-associated TB, sputum smear microscopy has now been replaced by Xpert MTB/RIF as the initial diagnostic test for TB [26].

Determine TB-LAM is a low-cost, point-of-care lateral-flow (‘strip test’) assay that diagnoses TB through detection in urine of lipoarabinomannan (LAM): a lipopolysaccharide component of the *M. tuberculosis* cell wall [28]. It has high specificity whereas sensitivity is very strongly CD4 count dependent, at best detecting approximately two-thirds of cases in those with CD4 counts <50 cells/μl [28-31]. This assay therefore allows rapid (<30 minutes) bedside diagnosis among those who have the highest mortality risk [32]. The growing evidence base on this assay will be reviewed by WHO in 2014. Its role is likely to be as an add-on test within the diagnostic algorithm to permit point-of-care diagnosis and immediate TB treatment among patients with advanced immunodeficiency (CD4 counts <200 cells/μl) following admission to hospital or enrolling in ART clinics [28,31].

#### Screening for HIV in those with TB or possible TB

A major step forward in improving HIV testing rates in patients with TB was the switch from voluntary counseling and testing (VCT) to provider-initiated testing and counseling (PITC) in 2007 [33]. With PITC, all patients undergo routine testing unless they specifically opt out. Testing has increased globally from 3.1% in 2004 to 40% of notified TB cases in 2011, but falls well short of the goal of universal testing [1]. Testing rates have reached 69% in Africa, >50% in the Americas and 32% in South-East Asia. In African countries, the proportion of TB patients testing positive is 46% overall (range, 8% to 77%) and exceeds 50% in ten counties in the south and east of the continent [1]. A further significant policy change has been to expand PITC to include all patients being investigated for TB regardless of whether or not TB is diagnosed [10,12]. This change resulted from the observed high HIV prevalence and mortality among those presenting for investigation of possible TB even when this diagnosis was subsequently excluded [34]. It is critical, however, that improved testing rates are accompanied by improvement in the delivery of appropriate management.

### Optimized TB treatment

The first priority for patients with HIV-associated TB is to immediately start effective TB treatment using a regimen containing RIF throughout [12,35]. A systematic review found that the incidence of relapse and/or failure among patients treated with intermittent (thrice weekly) TB therapy throughout was two to three times higher than that in patients who received a daily intensive phase [36]. Thus, the recommended optimum standard regimen is 2 months of rifampicin, isoniazid, pyrazinamide and ethambutol followed by 4 months of rifampicin and isoniazid (2HRZE/4HR), with therapy administered daily throughout [12]. Where this is not possible, an acceptable alternative is to use a thrice-weekly continuation phase. Treatment outcomes are worse for those with isoniazid monoresistance [36,37] and, thus, in settings with high prevalence of isoniazid monoresistance, 2HRZE/4HRE is the recommended first-line regimen [12]. Drug susceptibility testing is recommended to guide treatment in patients who have previously been treated for TB, although ideally all patients with TB should have drug susceptibility testing. Where the Xpert MTB/RIF assay is being rolled out as the primary TB diagnostic test, RIF resistance screening is now integral to the initial diagnostic process [26].

After several decades with no new advances in TB treatment, there are now some promising developments. For example, several large-scale phase III randomized controlled trials (including the ReMOX, Oflotub and RIFAQUIN studies) are evaluating whether incorporation of a newer fluoroquinolone into treatment regimens can be used to shorten treatment for drug susceptible TB [38]. The first of these to report, the RIFAQUIN study, found treatment shortening was associated with a higher rate of adverse outcomes including failure, relapse and death [39]. However, none of these studies have been designed to specifically address this question in HIV-infected clinical populations. There is also a growing developmental pipeline of new TB drugs, although these are most likely to be used in the treatment of MDR-TB, at least initially [38].

### Co-trimoxazole preventive therapy

Co-trimoxazole (trimethoprim sulfamethoxazole) is a low-cost, widely available and relatively safe antibiotic that reduces morbidity and mortality in people living with HIV due to prophylactic activity against a range of pathogens, including those causing bacterial sepsis, pneumocystis pneumonia, cerebral toxoplasmosis and malaria. Both observational and randomized controlled trials conducted in sub-Saharan Africa have shown that this simple intervention is associated with a substantial reduction in mortality among patients with HIV-associated TB (range, 19% to 46%) [40-44] (Table 4). This beneficial effect was observed in a range of settings with high or low rates of bacterial resistance to the drug and is additive in reducing early mortality when combined with ART [45].

**Table 4:** Impact of co-trimoxazole prophylaxis on mortality among predominately adult patients with HIV-associated tuberculosis (TB)

**Study**	**Year of publication**	**Study design**	**Country**	**Level of bacterial resistance to co-trimoxazole**	**No. of study participants**	**Mortality reduction**
Wiktor *et al*. [40]	1999	Randomized controlled trial	Cote D’Ivoire	Low	771	46%
Zachariah *et al*. [41]	2003	Cohort study (‘before’ and ‘after’ study with historical controls)	Malawi (north)	High	1,986	19%
Mwaungulu *et al*. [42]	2004	Cohort study (‘before’ and ‘after’ study with historical controls)	Malawi (south)	High	717	22%
Grimwade *et al*. [43]	2005	Cohort study (‘before’ and ‘after’ study with historical controls)	South Africa	High	3,325	29%
Nunn *et al*. [44]	2008	Randomized controlled trial	Zambia	High	1,003	21%

Routine administration of co-trimoxazole to patients with HIV-associated TB is recommended (480 mg twice per day or 960 mg once per day) [10-12]. Implementation of this simple, life-saving intervention has steadily increased from a negligible proportion in 2004 to 79% of all notified TB cases with a positive HIV test in 2011 (79% of those in the African region and 89% of those in the South-East Asian region) [1]. Coverage needs to increase to the 100% target set in the Global Plan to Stop TB, 2011-2015 [46]. Evidence is unclear as to whether co-trimoxazole should be continued indefinitely or might be discontinued once the CD4 cell count has reached a threshold of either 200 or 350 cells/μl [11]. The potential benefits of ongoing therapy may vary according to local factors such as the safety of the water supply, the presence of malaria and the local spectrum of opportunistic pathogens.

### Antiretroviral treatment

In observational cohort studies, concurrent ART reduces mortality risk by 64% to 95% in patients receiving treatment for HIV-associated TB [47]. In the South African Starting Antiretroviral Therapy at Three Points in Tuberculosis Therapy (SAPIT) randomized trial, receipt of concurrent ART was associated with survival benefit among those with CD4 cell counts of <200 cells/μl and 200 to 500 cells/μl [48]. Recommended first-line ART regimens for use with TB treatment are based on non-nucleoside reverse transcriptase inhibitors (NNRTI), with efavirenz (EFV) as the preferred choice and nevirapine (NVP) as an alternative. While first-line regimen choices are well established, second-line ART remains problematic. The recommended regimens and their pharmacokinetic interactions with TB treatment are shown in Table 5 and the hiv-druginteractions.org website provides a useful up-to-date source of information on interactions (see [49]). Combining the multidrug regimens used to treat TB and HIV is complicated not only by high pill burden and increased risks of drug-drug interactions, but also by cotoxicity and immune reconstitution inflammatory syndrome (IRIS).

**Table 5:** Approaches to cotreatment for HIV-infected patients with rifampicin-susceptible tuberculosis

**Combined regimens**	**Treatment recommendations**	**Drug-drug interactions**
Efavirenz + rifampicin-based TB treatment	No dose adjustments TDF + 3TC/FTC + EFV (WHO-recommended optimum regimen) AZT + 3TC + EFV (alternative WHO regimen)	Rifampicin induces CYP2B6 but inhibition of CYP2A6 by isoniazid might account for increased efavirenz concentrations during TB treatment in those patients with slow CYP2B6 metabolizer genotype
Nevirapine + rifampicin-based TB treatment	Omit 14 day lead-in phase of once daily dose of NVP TDF + 3TC/FTC + NVP (alternative WHO regimen) AZT + 3TC + NVP (alternative WHO regimen)	Rifampicin induces CYP2B6 and CYP3A4. Although TB treatment reduces nevirapine concentrations, toxicity concerns curtail increasing the dose and outcomes are acceptable (but inferior to EFV) on standard doses.
Lopinavir/ritonavir + rifampicin-based TB treatment	Double dose lopinavir/ritonavir (800/200 mg 12 hourly) Or superboost lopinavir (lopinavir/ritonavir 400/400 mg 12 hourly) Monitor alanine transaminase (ALT) closely.	Rifampicin induces CYP3A4, p-glycoprotein and OATP1B1. Ritonavir counteracts this effect and adjusted doses of ritonavir or lopinavir/ritonavir are used to compensate, but lopinavir concentrations may be more variable. Increased risk of hepatotoxicity, and gastrointestinal side effects.
PI/ritonavir + rifabutin-based TB treatment	Reduce rifabutin dose to 150 mg daily or thrice weekly. Monitor closely for rifabutin toxicity.	Ritonavir-boosted PIs markedly increase rifabutin concentrations and reduce its clearance necessitating reduction in the dose of rifabutin by 50% to 75%. Toxicity (neutropenia, uveitis, hepatoxicity, rash, gastrointestinal symptoms) and suboptimal rifamycin exposures with reduced dose are concerns.
Triple nucleoside/tide regimen + rifampicin-based TB treatment	No dose adjustments. A triple nucleoside/tide regimen should include tenofovir or abacavir. Monitor viral load.	Triple nucleoside/tide regimens may perform adequately in patients with viral suppression who have not failed a first line regimen, and provide alternative ART regimens in patients with contraindications to efavirenz or nevirapine, wehre other options are unavailable. TB treatment has minimal effect on tenofovir concentrations. Although rifampicin induces the enzymes responsible for glucuronidation of abacavir and zidovudine, this effect is not thought to be clinically important.

*3TC* 2′,3′-dideoxy-3′-thiacytidine, *ART* antiretroviral therapy, *CYP* cytochrome P450, *EFV* efavirenz, *FTC* emtricitabine, *OATP* organic anion-transporting polypeptide, *NNRTI* non-nucleoside reverse transcriptase inhibitor, *NVP* nevirapine, *PI* protease inhibitor, *TB* tuberculosis, *TDF* tenofovir, *WHO* World Health Organization.

#### Pharmacokinetic interactions with *fir*st-line ART

Although RIF induces the expression of cytochrome P450 2B6 (CYP2B6), which comprises the main metabolic pathway for EFV, studies have failed to demonstrate significantly reduced concentrations of EFV with concomitant RIF-based TB treatment [50-53]. This is consistent with the observed virological responses which are excellent in patients receiving RIF-based TB treatment treated with standard 600 mg daily doses of EFV [54-57] and were better than those in TB patients randomized to NVP-based ART in the recent CARINEMO trial [56]. Similarly, lowering the dose of EFV to 400 mg daily in the ENCORE1 trial did not compromise outcomes in non-TB patients [58]. Thus, although the US Federal Drug Administration (FDA) [59] recommends that the dose of EFV during RIF treatment is increased in adults weighing more than 50 kg, this is not supported by studies in TB patients [53] and is not recommended by the WHO for resource-limited settings.

Conversely, however, among patients with a slow *CYP2B6* metabolizer genotype, EFV concentrations are increased during TB treatment, possibly due to inhibition by INH of accessory pathways metabolizing EFV [60,61]. This genotype is relatively common in Africa, South-East Asia, and the Caribbean [50-52,62,63]. Whether EFV-induced central nervous system (CNS) adverse effects are more frequent during TB treatment or isoniazid preventive therapy in patients with this genotype needs to be evaluated.

NVP is a reasonably safe, acceptable alternative for TB patients unable to tolerate EFV. Through induction of the expression of CYP2B6, RIF treatment reduces NVP concentrations by an average of approximately 40% and NVP-based ART remains inferior to EFV-based regimens in TB patients [56]. During the 14-day lead-in phase of NVP dosing, plasma drug concentrations are very low in patients receiving RIF, potentially predisposing to the development of viral resistance mutations and contributing to an increased risk of virological failure [54]. The CARENIMO trial recently found that NVP was well tolerated when introduced at full doses (200 mg twice a day) in patients with CD4 cell counts <250 cells/mm^3^ receiving RIF [56]. The use of a dose escalation lead-in phase to avoid toxicity in patients receiving RIF is therefore not recommended.

Triple nucleoside/tide regimens are less effective than NNRTI-based or PI-based regimens particularly in patients with baseline viral loads >100,000 copies/ml [64]. However, small uncontrolled studies suggest they may provide an acceptable regimen for TB patients who have not failed an ART regimen [65,66] even though the concentrations of abacavir and zidovudine may be reduced by concomitant RIF. This therefore provides an alternative option for those in whom EFV and NVP are contraindicated and integrase inhibitors unavailable.

#### Pharmacokinetic interactions with *seco*nd-line ART

With increasing numbers of patients switching to protease inhibitor (PI)-based second-line ART regimens, defining safe and effective approaches to concurrent TB treatment is an urgent challenge. The pharmacokinetic interactions between rifamycins and PIs are extensive. RIF reduces concentrations of ritonavir-boosted PIs by 75% to 90% [67]. Conversely, through potent inhibition of CYP3A4 and p-glycoprotein, high-dose ritonavir offsets the effect of RIF-mediated induction such that ‘superboosting’ of lopinavir or saquinavir (lopinavir/ritonavir 400 mg/400 mg or saquinavir/ritonavir 400 mg/400 mg, twice daily) preserves plasma concentrations of the PI [68-70]. Adequate plasma concentrations of lopinavir are also achieved in adults by doubling the dose of lopinavir/ritonavir in the tablet formulation (to 800/200 mg twice daily); this is the simplest approach, especially in settings where the separate ritonavir is not available [71]. Although these approaches are associated with high rates of hepatotoxicity in studies of healthy volunteers, these seem to be much safer in HIV infected patients [71-76]. Nevertheless, hepatotoxicity, gastrointestinal side effects and poor tolerability are problematic and treatment discontinuation rates of up to nearly 50% have been reported [74,75].

Rifabutin is an alternative rifamycin to RIF, but data on its use in TB patients receiving ritonavir-boosted PIs are limited. Studies of healthy volunteers show that ritonavir-boosted PIs increase the concentrations of rifabutin approximately fourfold and the concentrations of the active metabolite to an even greater extent. Thus, the dose of rifabutin needs to be reduced. Thrice weekly 150 mg doses of rifabutin in combination with standard doses of lopinavir/ritonavir may be reasonably tolerated [77,78]. However, contrary to expectations based on pharmacokinetic data from healthy volunteers, small studies in coinfected patients have found that rifabutin 150 mg used thrice weekly in combination with lopinavir/ritonavir resulted in low rifabutin concentrations [79-82]. Such levels would be conducive to acquisition of rifamycin resistance in patients with severe immunosuppression [79,83] as has been observed with twice weekly doses [84]. Thus, recent US national guidelines recommend a daily 150 mg dose of rifabutin for patients on ritonavir-boosted PIs [85].

There is extremely limited information about the safety or efficacy using rifabutin with PIs and this may vary between populations due to differential increases in rifabutin concentrations. Severe neutropenia and uveitis occur relatively frequently in patients with increased exposures [81,86] and hepatitis, gastrointestinal symptoms, rashes and anemia are also important safety concerns [87,88]. While rifabutin is becoming more widely available and affordable, it is not an ideal solution for high burden settings where limited patient monitoring is available and fixed dose drug formulations are preferred. Thus, there is an urgent need for research to define the optimum approaches for the cotreatment of patients with TB who have failed first-line ART, including the use of newer agents.

#### Pharmacokinetic interactions with newer ART drugs

Ritonavir-boosted darunavir has a favorable safety and tolerability compared to lopinavir/ritonavir and promising efficacy, especially in treatment of ART-experienced patients. A pharmacokinetic study in healthy volunteers suggests that it could be used in standard doses with rifabutin 150 mg thrice weekly, but the drug-drug interactions with RIF have not been studied. Integrase inhibitors have potent antiviral activity and are well tolerated, but any future role in ART programs in low-resource settings is at present undefined. However, initial data on use with TB treatment show promise. Pharmacokinetic studies suggest that doubling the dose of raltegravir to 800 mg twice daily compensates for the effect of RIF on overall exposure [89,90] and this approach seems to be well tolerated and effective in patients with HIV-associated TB [91]. However, preliminary results of the REFLATE TB study suggest that such dose adjustment may not even be necessary as virological responses were similar in ART-naive TB patients receiving RIF who were randomized to receive 400 mg or 800 mg of raltegravir twice daily or EFV daily [92]. Similar to raltegravir, a pharmacokinetic study of dolutegravir in healthy volunteers suggests that the effect of RIF on antiretroviral therapy can be overcome by increasing the daily 50 mg dose of dolutegravir to 50 mg twice daily and that dose adjustment may not be necessary with rifabutin [93].

#### Timing of ART initiation during TB treatment

The optimum time to start ART in patients with HIV-associated TB is subject to a complex series of competing risks [94] and must balance the high risk of morbidity and mortality in patients with very low CD4 cell counts and severe disease with the potential occurrence of additive toxicities and immune reconstitution inflammatory syndrome (IRIS). Results of large randomized strategy trials are now available to inform guidelines (Table 6) [48,55,95-98]. Patients with baseline CD4 counts of <200 and 200 to 500 cells/μl have improved survival benefit from coadministered ART [48] and WHO recommends that ART be given to all patients concurrently with TB treatment regardless of the CD4 count. Trial data also demonstrated that mortality was reduced in those with the most severe immunodeficiency (CD4 cell counts <50 cells/μl) if they stated ART within the first 2 weeks of TB treatment [11]. For patients with less severe immunosuppression (CD4 counts >50 cells/μl), data suggested that ART might be deferred until completion of the intensive phase of TB treatment without compromising survival but reducing the risk of morbidity from TB-IRIS [55,96].

**Table 6:** Randomized controlled studies of the timing of starting antiretroviral therapy (ART) during tuberculosis (TB) treatment

**Study**	**Study population**				**Methods**		**Results**			
	**N**	**Location**	**TB**	**Median CD4+ cells/mm**^**3 **^**(IQR)**	**Timing of ART in weeks ‘earlier’ vs ‘later’**	**Primary endpoint**	**Follow-up in months**	**Primary endpoint ‘earlier’ vs ‘later’**^**a**^	**Primary endpoint in CD4 <50 cells/μl**^**b**^	**TB immune reconstitution**
SAPIT [48] (first analysis)	429	South Africa	Smear-positive pulmonary TB	150 (77 to 254)	<12 vs after end TB treatment	Death	12.1	5.4 vs 12.1 *P* = 0.003^c^	Not reported	12.4% vs 3.8% *P* <0.001
SAPIT [96] (second analysis)	429	South Africa	Smear-positive pulmonary TB	150 (77 to 254)	Within 4 vs 8 to 12	AIDS or death	17.7	6.9 vs 7.8 *P* = 0.73	8.5 vs 26.3^b^*P* = 0.06	20.1% vs 7.7% *P* <0.001
CAMELIA [95]	660	Cambodia	Smear-positive TB	25 (11 to 56)	2 vs 8	Death	25	18% vs 27%, *P* = 0.006	Not reported^d^	33.1% vs 13.7% *P* <0.001
STRIDE [55]	809	Multicontinent^e^	Confirmed or presumed pulmonary or extrapulmonary TB	77 (36 to 145)	2 vs 8 to 12	AIDS or death	12	12.9% vs 16.1% *P* = 0.45	15.5% vs 26.6% *P* = 0.02	11% vs 5% *P* = 0.02
TB Meningitis [97]	253	Vietnam	TB meningitis	39 (18 to 116)	≤1 vs 8	Death^f^	12	59.8% vs 55.6% *P* = 0.50	63.3% vs 65.1% *P* = 0.84	Not reported
TIME Trial [98]	156	Thailand	Confirmed or presumed pulmonary or extrapulmonary TB	43 (37 to 106)	4 vs 12	Death	96 weeks	7.6% vs 6.5% *P* >0.99	8.7% vs 13.1% *P* = 0.725	8.86 vs 5.02 *P* = 0.069

Footnotes:

^a^Presented either as cumulative incidence of primary endpoint in early vs. later arm (%) or as events per 100 person-years.

^b^Prespecified analysis.

^c^Significant difference in mortality observed in patients with either CD4 counts <200 cells/μl or 200 to 500 cells/μl.

^d^Lower CD4 was not associated with an increased risk for the primary endpoint.

^e^North America, South America, Asia, Africa.

^f^Primary endpoint was all cause mortality at 9 months.

WHO guidelines reflect these findings, recommending that TB treatment should be started first and followed by ART as soon as possible within the first 8 weeks of treatment but within the first 2 weeks for those with profound immunosuppression (CD4 count <50 cells/μl) [11]. However, CD4 count measurements may either be unavailable or be inaccurate in some settings. In addition, within different CD4 count categories, there is great diversity in severity of disease and mortality risk. Thus, where feasible, decisions on timing for individual patients might also be further informed by taking into account clinical criteria such as body mass index, Karnofsky score, severity of anemia and extent of TB. Moreover, national guidelines might best be appropriately tailored for operational simplicity. One possible option, for example, might be to start ART in all patients after 2 weeks of TB treatment, accepting lower risk of mortality but higher risk of TB IRIS.

Patients with HIV-associated TB meningitis represent an important exception. A randomized trial from Viet Nam found no survival benefit from early ART in patients with TB meningitis [97], reflecting the awful prognosis (mortality approximately 60%) of these patients with advanced disease and the dire consequences of TB-IRIS within the confined space of the CNS [99]. Further studies are required in different geographical settings to better define appropriate management of these patients.

#### Adverse drug reactions and management

Antituberculosis and antiretroviral drugs have overlapping toxicity profiles that include drug-induced liver injury (DILI), cutaneous reactions, renal impairment, neuropathy and neuropsychiatric adverse effects (Table 7). These complicate management in a substantial minority of patients.

**Table 7:** Shared side effects of antiretroviral therapy (ART) and antituberculosis drugs

**Adverse effects**	**Antiretroviral drugs**	**Antituberculosis drugs**
Gastrointestinal disturbance and/or pain	AZT, ddI, PIs	RIF, INH, PZA, ethionamide, PAS, clofazamine, linezolid
Liver injury	NVP, EFV, PIs, NRTIs^a^	RIF, INH, PZA and many second line drugs including ethionamide, fluoroquinolones, PAS
Peripheral neuropathy	D4T, ddI	INH, ethionamide, terizidone/cycloserine, linezolid
Neuropsychiatric	EFV	Terizidone/cycloserine, ethionamide, fluoroquinolones, INH
Renal impairment	TDF	Aminoglycosides and capreomycin
Rash	NVP, EFV, ABC	Rifampicin, INH, PZA, ethambutol, streptomycin and many second line drugs including fluoroquinolones, PAS, clofazamine
Blood dyscrasias	AZT, 3TC	Linezolid, rifabutin, INH, rifampicin
Cardiac conduction abnormalities	PIs	Bedaquiline, fluoroquinolones, clofazamine
Pancreatitis	D4T, ddI	Linezolid
Lactic acidosis	D4T, ddI	Linezolid

*3TC* 2',3'-dideoxy-3'-thiacytidine, *ABC* abacavir, *AZT* zidovudine, *D4T* stavudine, *ddI* didanosine, *EFV* efavirenz, *INH* isoniazid, *NRTIs* nucleoside reverse transcriptase inhibitors, *NVP* nevirapine, *PAS* para-aminosalicylic acid, *PIs* protease inhibitors, *PZA* pyrazinamide, *RIF* rifampicin, *TDF* tenofovir.

^a^NRTIs (especially D4T and ddI) can cause steatohepatitis.

In patients without coinfection, DILI (variably defined as, for example, an elevation of alanine aminotransferase to >3 or >5 times the upper limit of the normal range) occurs in 5% to 33% of those receiving TB treatment [100] and in 5% to 11% of those receiving currently recommended ART regimens [101,102]. HIV infection itself has been identified as a risk factor for DILI in patients receiving TB treatment in some [103,104] but not all studies [105-108]. Of the currently used ART drugs, NVP is associated with highest risk of DILI; however, EFV and PIs are also recognized causes.

Concurrent TB treatment in patients receiving NNRTI-based ART has been associated with an increased risk of DILI in some [109-111] but not all [54] studies. In one of these, the absolute risk of severe hepatotoxicity in patients receiving EFV-based ART was low, but the risk associated with concurrent TB treatment exceeded that associated with positive hepatitis B surface antigen status [109]. Importantly, a randomized trial of NVP-based versus EFV-based ART in patients receiving TB treatment reported more treatment discontinuations related to DILI in the NVP arm (4 vs 0%) [56].

Development of DILI significantly complicates management of HIV-associated TB. Elevation of alanine transaminase (ALT) concentrations >3 to 5 times the upper limit of normal especially when accompanied by symptoms or jaundice requires that all potentially hepatotoxic medication is interrupted until derangements of liver function tests resolve. Thereafter, rechallenge of first-line TB medication should be considered followed by ART, although rechallenge is generally not undertaken if there was liver failure. Rechallenge strategies have not been studied in randomized trials in HIV-infected patients. However, in the largest randomized trial of TB without HIV coinfection, approximately 90% of patients were rechallenged with their first-line TB drugs without recurrence [112]. Risk of recurrence was not related to whether the four first-line TB drugs were reintroduced sequentially or concurrently. Further studies are needed to define the optimum rechallenge strategy in coinfected patients in whom both TB treatment and ART require reintroduction. Until further evidence emerges, the American Thoracic Society recommends that RIF can be reintroduced in coinfected patients once the ALT is less than two times the upper limit of normal followed by reintroduction of INH with monitoring of liver function [100]. However, they also suggest that pyrazinamide is not reintroduced.

While some cohort studies have suggested low morbidity and mortality in HIV-infected patients with DILI [109], mortality is substantial among those requiring hospital admission. In a South African study, mortality was 35% among patients admitted to hospital with DILI during TB treatment, ART or concurrent therapy [113]. Reasons for these deaths were sepsis and liver failure, although interruption of required TB treatment and ART are likely to have played a role.

TB treatment is associated with a spectrum of cutaneous adverse reactions including morbiliform rashes, Steven Johnson syndrome and toxic epidermal necrolysis, fixed drug eruption, lichenoid drug eruptions and acute generalized exanthematous pustulosis [114]. Cotrimoxazole, NVP, and to a lesser extent EFV, can also cause many of the same clinical presentations [102,115,116]. HIV coinfection was associated with a fivefold increased risk of rash or drug fever in one study [117] but small, non-significant increases in risk in others [105,108]. If a clinically significant rash develops, all potentially responsible drugs need to be interrupted and then a carefully monitored rechallenge of first-line TB drugs can be considered once the rash has resolved. In a cohort of mainly HIV-infected patients rechallenged following cutaneous reactions to TB drugs, 50% developed reintroduction reactions but only a small minority were severe [118].

Renal dysfunction may be caused via different mechanisms in patients receiving tenofovir, RIF or aminoglycosides (used for MDR-TB). Tenofovir and aminoglycosides may both cause tubular cell toxicity at the level of the proximal renal tubules, whereas RIF infrequently causes a tubulointerstitial nephritis mediated by immune hypersensitivity. Case reports describe renal failure in patients receiving a combination of tenofovir and aminoglycosides, although cohort studies have not confirmed an increased risk [119]. The combination is best avoided when possible. In patients with significant renal dysfunction, Use of tenofovir should be avoided where possible and dosing of ethambutol, NRTI drugs, some quinolones (ofloxacin and levofloxacin) and certain other second-line antituberculosis drugs (including cycloserine, para-aminosalicylic acid, clofazamine and linezolid) needs to be adjusted*.*

#### TB Immune reconstitution inflammatory syndrome (IRIS)

Two major forms of TB immune reconstitution syndrome (TB-IRIS) are recognized and these are called paradoxical TB IRIS and unmasking TB-IRIS and case definitions have been published [120]. Paradoxical TB-IRIS is an important cause of morbidity in patients known to have HIV-associated TB and occurs within the first weeks of ART [120,121]. The typical clinical course of paradoxical TB-IRIS is as follows. Initiation of TB treatment in a patient with HIV infection and newly diagnosed TB results in clinical stabilization or improvement. However, subsequent introduction of ART is accompanied by recurrence or exacerbation of TB symptoms with new or worsening clinical signs of TB that often have a marked inflammatory component [120,121].

While seldom life-threatening, deaths due to paradoxical TB-IRIS have been described. Two major risk factors identified in observational studies [122-125] and in clinical trials [55,95,126] are a low CD4 count prior to ART and a shorter interval between starting TB treatment and ART. There is no diagnostic test for TB-IRIS; the diagnosis is based on clinical presentation and exclusion of alternative diagnoses such as bacterial infection or drug resistant TB [120]. However, drug-resistant TB is not only in the differential diagnosis as an alternative cause of the clinical deterioration but may also be a risk factor for the development of paradoxical TB-IRIS [127].

The second major form of TB-IRIS is commonly referred to as ‘unmasking’ TB-IRIS. This occurs when active TB is present but remains undiagnosed at the time of starting ART [120,128]. Subsequent immune recovery triggers the overt symptomatic presentation of TB. In a proportion of cases, unusual inflammatory features may also develop and such cases are regarded as having ‘unmasking’ TB-IRIS. Risk of unmasking TB-IRIS is therefore directly related to the efficiency of the pre-ART screening process and the resulting prevalence of undiagnosed disease.

Both types of TB IRIS have a wide range of clinical features often with involvement of multiple organ systems, reflecting widespread dissemination of *M. tuberculosis* in those with profound immunosuppression. Common features include fever, recurrence of respiratory symptoms with worsening infiltrates on chest radiographs, enlargement of lymph nodes (often with suppuration), formation of tuberculous abscesses and serous effusions [120,121]. There are many case reports of unusual and diverse complications, including granulomatous nephritis with renal impairment, parotitis, epididymo-orchitis, granulomatous hepatitis, splenic enlargement and abscess formation, psoas abscess, peritonitis, ascites and intestinal involvement [120,121]. Neurological TB-IRIS is particularly severe, manifesting with tuberculomas, tuberculous abscesses, cerebral edema, meningitis and radiculomyelopathy [99,129,130]. Neurological TB IRIS has a much poorer outcome compared to other forms, with a mortality of 13% to 75% [99,129,130].

In most cases, the onset of paradoxical TB-IRIS is within the first 4 weeks of ART (median 14 days (IQR, 8 to 23) in 1 series [127]) but can occur within a few days. The proportion of patients affected ranges widely from 0% to over 40% [120] and this may relate to differences in risk factors and case definitions. In a meta-analysis, the summary risk estimate was 15.7% [131]. Of these, 3.2% died, representing approximately 1 in 200 patients with HIV-associated TB who start ART. The median duration of TB-IRIS symptoms has been reported to be 2 to 3 months [124,125] but a minority of cases have a protracted course which may last for more than 1 year [120,124,132]. Such protracted cases typically have persistent or recurrent suppurative lymphadenitis or abscess formation. However, the majority of cases have a favorable long-term outcome [133].

TB-IRIS is not an indication for stopping ART, although this should be considered in life-threatening cases such as those with cerebral edema and depressed level of consciousness or severe respiratory failure. In mild cases, no specific treatment is usually required; the patient should be treated symptomatically and counseled regarding the need to continue ART and TB treatment. Corticosteroids should be considered if symptoms are more significant. In a randomized placebo-controlled trial, prednisone used at a dose of 1.5 mg/kg/day for 2 weeks followed by 0.75 mg/kg/day for 2 weeks was associated with reduced morbidity (duration of hospitalization and need for therapeutic procedures) [134]. Symptom improvement was more rapid and there was no excess risk of other severe infections [134]. Although no mortality benefit was demonstrated, patients with immediately life-threatening TB-IRIS were not enrolled in view of ethical considerations. Indeed, most experts recommend steroid therapy for life-threatening TB-IRIS, especially IRIS involving the CNS. A subgroup of patients in this trial (approximately one in five) relapsed after stopping prednisone and required a further and more prolonged course to control symptoms [134]. Similarly, in other settings, TB-IRIS has relapsed in up to 50% of patients after stopping steroids [133] and thus the duration of therapy must be tailored according to the clinical response.

Non-steroidal anti-inflammatory drugs (NSAIDs) have also been used in the treatment of TB IRIS although no clinical trial data exist to support their use. Other forms of immunomodulatory therapy such as thalidomide, azathioprine and tumor necrosis factor α blockers (such as adalumimab) have been used in cases refractory to steroid therapy with anecdotal reports of benefit [135]. In patients with suppurative lymphadenitis or abscesses, needle aspiration may provide a pus sample to exclude drug-resistant TB as well as bringing symptomatic relief.

There is no evidence base for pharmacological prevention of TB-IRIS. However, this needs to be considered in view of the recommendation within guidelines for early ART initiation in TB patients with advanced HIV [11]. Adjunctive immunomodulatory therapies might reduce the risk or severity of TB-IRIS in such patients. A randomized placebo-controlled trial of prednisone for prevention of TB-IRIS in high-risk patients (CD4 counts <100 cells/mm^3^ starting ART within 30 days of TB treatment) is underway [136]. Until results from this trial are available corticosteroids cannot be recommended for prevention of TB IRIS with the exception of patients with TB of the CNS for whom adjunctive steroids form part of the standard of care [137]. However, in such patients, TB IRIS occurs in approximately 50% of patients with CNS TB starting ART despite receipt of corticosteroids [99].

Other agents that have been proposed for prevention of TB IRIS are vitamin D, statins and the C-C chemokine receptor type 5 (CCR5) blocker maraviroc [135]. Vitamin D has modulating effects on both the adaptive and innate immune responses [138,139]. Statins have anti-inflammatory properties and there is precedence for using these agents for autoimmune inflammatory disorders in an experimental model [140,141]. However, neither vitamin D nor statins have yet been tested in clinical studies. Maraviroc, however, was shown not to prevent IRIS in a placebo-controlled trial conducted in Mexico and South Africa [142].

### Management of HIV-associated MDR-TB

The emergence of MDR-TB and extensively drug resistant TB (XDR-TB) has compounded the HIV-associated TB epidemic in resource-limited settings [5,143]. MDR-TB is caused by strains that are resistant to both rifampicin and isoniazid whereas XDR-TB strains are MDR-TB strains with additional resistance to any quinolone drug and any one of the second-line injectable aminoglycosides (amikacin, capreomycin or kanamycin). Much disease remains undiagnosed due to lack of laboratory capacity. However, increasing implementation of the Xpert MTB/RIF assay now provides the means for rapid screening for RIF resistance, although follow-on testing is then required to further characterize the full drug susceptibility pattern. This can be performed phenotypically through culture-based systems but is very slow. In 2008, WHO approved the use of line probe assays for the rapid molecular detection of drug resistance in smear-positive specimens or culture isolates [144] and a range of commercially available assays now offer the possibility of much more rapid diagnosis of both MDR-TB and XDR-TB [145]. However, line-probe assays can only be used where appropriate laboratory facilities and expertise exist as they are highly technically demanding and are well beyond the scope of most resource-limited settings apart from in specialized reference laboratories.

Worldwide, successful treatment of MDR-TB is achieved in only approximately 50% to 60% of patients [146,147], but management is considerably more difficult in resource-limited settings and especially in those with HIV coinfection due to late diagnosis with more frequent extrapulmonary dissemination, high risks of drug cotoxicity and IRIS, copathology and poor adherence with prolonged, toxic regimens. The WHO recommends that patients with confirmed MDR-TB should receive a regimen containing pyrazinamide together with at least four second-line drugs in the intensive phase that are likely to be effective, including a fluoroquinolone (using a later generation agent where possible), a parenteral agent (such as amikacin or kanamycin), ethionamide (or prothionamide) and either cycloserine or p-aminosalicylic acid (PAS) [13]. An intensive phase of 8 months and a total treatment duration of 20 months is suggested for most patients, but may be modified according to response. A range of other second-line drugs that have limited efficacy may be used for treatment of XDR-TB and treatment regimens should be based upon drug susceptibility testing [13]. However, evidence to inform best practice is lacking and outcomes are often poor.

Co-trimoxazole prophylaxis and ART are recommended for all patients with HIV-associated MDR-TB regardless of CD4 count and the timing of ART initiation is similar as for drug-susceptible TB [11]. Many of the second-line MDR-TB drugs are poorly tolerated and drug discontinuation rates are high as a result of adverse effects. MDR-TB may be a risk factor for TB IRIS in view of slow mycobacterial antigen clearance [127]. Nutritional depletion and co-morbid conditions may further undermine outcomes.

Adverse events are frequent in HIV-infected patients receiving MDR treatment, the most common being gastrointestinal symptoms, peripheral neuropathy, hypothyroidism, deafness, psychiatric symptoms and hypokalemia [148,149]. In up to 40% of patients these adverse events are severe [148]. This relates to the inherent toxicity associated with MDR drugs; it does not appear that HIV-infected patients experience a higher incidence of adverse events than HIV-uninfected patients, nor that coadministration with ART increases toxicity [148,150,151].

Antiretroviral drugs do share common toxicities with second-line antituberculosis drugs, however (Table 7). Some of the most challenging of these are neuropsychiatric side effects. EFV causes inattention, vivid dreams and dizziness in up to 50% of patients, but in a minority these can be severe with mood disturbance or psychosis. Cycloserine (or terizidone) is a well recognized cause of psychosis, seizures and other CNS side effects although several other drugs such as the quinolones, ethionamide and high dose isoniazid can also cause CNS side effects. If patients develop severe CNS side effects it may be necessary to withdraw all possible culprit drugs with careful sequential reintroduction once resolved. Cycloserine should probably be regarded as the most likely culprit for psychosis and seizures. Antipsychotic or antidepressant medications may be required. EFV should not be routinely avoided because the majority of MDR-TB patients tolerate it well.

Much research is needed on how to improve treatment for drug-resistant TB. A shortened MDR-TB regimen of 9 months, which was found to be effective and well tolerated in Bangladesh [152], is now being evaluated in Ethiopia, South Africa and Vietnam and includes patients with HIV-associated TB. In the future, the newly approved agent bedaquiline (TMC-207) as well as two new nitroimidazoles (PA-824 and delaminid (OPC67683) under evaluation) may offer the prospects of improved treatment for MDR-TB [38]. However, a prolonged timeline is needed to adequately define how to combine existing agents and new drugs in regimens that optimize outcomes and that can be combined with ART in those with HIV-associated TB.

## Conclusions

The HIV-associated TB epidemic is a major challenge to international public health, remaining the most important opportunistic infection in people living with HIV globally and accounting for nearly 0.5 million deaths each year. However, over the past 10 years, major progress has been achieved in defining guidelines for the optimum case management with a combination of co-trimoxazole prophylaxis, optimally timed ART, and diagnosis and appropriate supportive care for treatment complications including drug toxicity and IRIS. The major remaining challenges are the management of TB in the increasing proportion of patients receiving PI-containing ART and the management of drug resistant TB. Having defined case management strategies, the ongoing challenge is to further develop effective, comprehensive and sustainable means of delivery through health systems.

## Abbreviations

ALT: alanine transaminase; ART: antiretroviral treatment; CNS: central nervous system; CYP: cytochrome P450 enzyme; E: ethambutol; EFV: efavirenz; H/INH: isoniazid; IRIS: immune reconstitution inflammatory syndrome; LAM: lipoarabinomannan; MDR-TB: multidrug resistant tuberculosis; NNRTI: non-nucleoside reverse transcriptase inhibitor; NVP: nevirapine; PI: protease inhibitor; PITC: provider initiated counseling and testing; R/RIF: rifampicin; TB: tuberculosis; VCT: voluntary counseling and testing; WHO: World Health Organization; XDR-TB: extensively drug resistant tuberculosis; Z: pyrazinamide.

## Competing interests

The authors declare they have no competing interests.

## Authors’ contributions

The first draft was written by SDL, GM and HMcI. All authors contributed to the development of subsequent and final drafts. All authors approved the final version.
